# Assessing the Influence of Socioeconomic Status and Air Pollution Levels on the Public Perception of Local Air Quality in a Mexico-US Border City

**DOI:** 10.3390/ijerph17134616

**Published:** 2020-06-27

**Authors:** Dalia M. Muñoz-Pizza, Mariana Villada-Canela, M. A. Reyna, José Luis Texcalac-Sangrador, Jesús Serrano-Lomelin, Álvaro Osornio-Vargas

**Affiliations:** 1 Doctorado en Medio Ambiente y Desarrollo, Instituto de Investigaciones Oceanológicas, Universidad Autónoma de Baja California, Ensenada 22860, Mexico; 2 Cuerpo académico de Bioingeniería y Salud Ambiental, Universidad Autónoma de Baja California, Mexicali 21100, Mexico; mreyna@uabc.edu.mx; 3 Environmental Health Department, Center for Population Health Research, National Institute of Public Health, Ciudad de Mexico 14080, Mexico; jtexcalac@insp.mx; 4 Department of Obstetrics & Gynecology, Heritage Medical Research Centre, University of Alberta, Edmonton, AB T6G 2R7, Canada; jaserran@ualberta.ca; 5 Department of Pediatrics, Faculty of Medicine & Dentistry, University of Alberta, Edmonton, AB T6G 1C9, Canada; osornio@ualberta.ca

**Keywords:** air pollution, social vulnerability, PM_10_, perception, Mexicali, sustainable development

## Abstract

Air pollution in developing countries is a growing concern. It is associated with urbanization and social and economic structures. The understanding of how social factors can influence the perception and the potential impact of air pollution have not been addressed sufficiently. This paper addresses the social vulnerability and exposure to PM_10_ association and its influence on the air quality perception of residents in Mexicali, a Mexico–US border city. This study used individual variables and population census data, as well as statistical and spatial analyses. A cluster of socially vulnerable populations with high exposure to coarse particulate matter (PM_10_) was found in the city’s peripheral areas. The spatial distribution of the local perception of air quality varied by the exposure zones of the estimated PM_10_ concentrations. Respondents living in very high exposure areas perceive air quality as “poor,” contrarily to a worse perception in areas of intermediate and lower exposure to PM_10_. Proximity to stationary sources of pollution was associated with a poor perception of air quality. Results also indicate that low household income and poor air quality perceived at the place of residence negatively influences the perceived changes in the air quality over time. The knowledge of chronic health effects related to air pollution was scarce in the sampled population, especially in the areas with very high exposure and high social vulnerability. These findings can serve as a support in local air quality management.

## 1. Introduction

### 1.1. Air Pollution in the Context of Sustainable Development

Air pollution is recognized as a threat to public health with significant social, economic and material consequences [1]. Ambient air pollution, mainly due to fine particulate matter (PM_2.5_) has been associated with three million deaths annually from no communicable diseases [2]. It mainly affects the quality of life and the economy of people living in low-middle income countries [3,4]. This issue has an essential relationship with the Sustainable Development Goals (SDGs). In particular, air pollution is linked to Goal 3 (good health and well-being), Goal 7 and 9 (clean energy, industry innovation and infrastructure) and Goal 11 (sustainable cities and communities) [5]. It also contributes indirectly to Goal 1, related to no poverty, building the resilience of people in vulnerable situations and reducing their vulnerability to economic, social, and environmental impacts [6]. A representation of such relationships is found in developing countries where the rapid urbanization process has led to a widening of the economic inequalities, growth with disordered changes in land use, industrial expansion, and a noticeable deterioration of the air quality [7,8,9]. High concentrations of particulate matter (PM_10_ and PM_2.5_) remain a concern in low-and middle-income communities in several countries of Latin America due to the existing levels exceeding the World Health Organization Air Quality Guidelines (an annual mean of 20 µg/m^3^ for PM_10_ and 10 µg/m^3^ for PM_2.5_) [4,10]. The high levels of particulate matter in Chile and the upper middle-income nations of Brazil and Mexico have been associated with an increase in respiratory and cardiorespiratory morbi-mortality risk, where people living in lower socioeconomic groups are at a higher risk [11,12,13]. There is evidence supporting the role of socioeconomic status as a significant effect modifier of air pollution-related health outcomes in disadvantaged populations [14,15,16]. However, the evidence is still scarce in Latin American countries [17], and consequently, there is an even a scarcer number of public policy initiatives [18]. Public policy programs to mitigate air pollution are widely supported by the technical information of the sources, levels and health issues. Factors related to social vulnerability (the capacity of individuals or communities exposed to environmental hazards to resist, cope with, and recover from these hazards [19]) have received less attention. Social vulnerability is a complex concept and involves socioeconomic status, demographic factors, perception, social networks, access to infrastructure, and political power [20]. However, when addressing air pollution, it has been overlooked, despite its relevance in sustainable development [21]. Policies to improve air quality represent opportunities to advance in SDGs [22,23], especially if they integrate relationships between social, economic and environmental factors. In this sense, effective policies to control air pollution with a broader socioeconomic impact require the understanding of the factors that influence the social vulnerability and the perception of air quality as evidence needed in the communication and implementation of appropriated strategies [24].

### 1.2. Air Quality Perception

Air quality public perception is a relevant input for evidence-based decision making. It influences the behavioral response to interventions and the choices that can lead to increasing or reducing exposure to air pollutants [25,26], as well as to new technological developments and regulatory strategies. Furthermore, public perception may be influenced by trust in institutions and socioeconomic characteristics such as income, education and age, and by contextual factors such as proximity to fixed sources of pollution or the presence of green areas in neighborhoods [27,28].

Studies conducted in Nanchang, Shanghai, and Wuhan (China) [24,29,30], southern Chilean cities [31], Colombian cities [32] and Mexico City [33] have demonstrated the importance of such socioeconomic and geographical factors in the decision making and implementation of air quality control strategies. Despite these efforts, there is a gap in the knowledge of the relationships among socioeconomic disparities, environmental degradation, and public perception [34]. This situation requires addressing in order to support air quality policies in the context of sustainable development. In this paper, we evaluated patterns of PM_10_ exposure levels, air quality perception, and social vulnerability in Mexicali, a representative city of the social and environmental problems present at the Mexico–US border region. Mexicali is a city affected by problems such as a marginalized migrant population and low-income communities exposed to multiple risks related to water and air pollution [35,36].

Explicitly, we modelled and characterized a city-wide exposure to PM_10_. PM_10_ concentrations compromise the air quality in Mexicali city. In addition to its geological origin, PM_10_ levels also reflect other persistent problems in the region. Local sources of PM_10_ relate to deficient urban solid waste management, agricultural waste burning, increased road traffic (abrasion, erosion, and exhaust emissions), resuspension from unpaved streets, and emissions from maquiladoras [37,38]. We developed a socioeconomic index to evaluate the spatial patterns of social vulnerability related to PM_10_ exposure, and we evaluated the association between the spatial distribution of the population’s air quality perception and the estimated exposure. All these were to analyze the factors that influence air quality perception using data from surveys and previous estimations of exposure and social vulnerability. These analyses seek to provide policy-relevant evidence to local air quality management in the context of sustainable development goals.

## 2. Methods

Exposure patterns to PM_10_ across the city were developed using 2015 PM_10_ data, a socioeconomic index was built on 2010 Census data, and the air quality perception at the individual level in inhabitants of Mexicali was approached by a survey performed in 2019. A detailed description of the methods is found below.

### 2.1. Study Site

**Figure 1:**
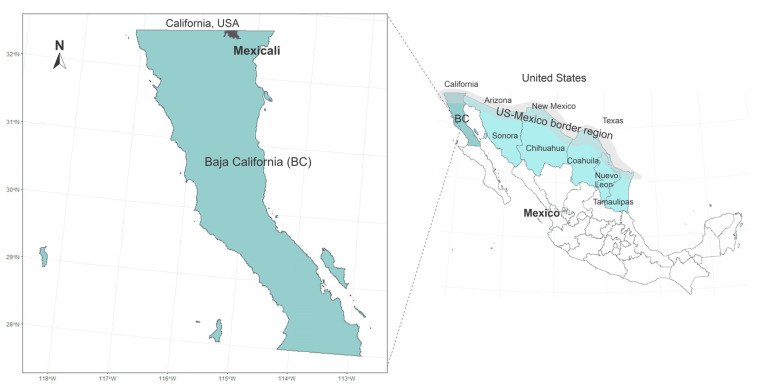
The geographic location of Mexicali within the US–Mexico border region.

More than 15 million inhabitants live in the US–Mexico borderlands [39]. This region is affected by poverty, marginalization, social inequities, and crucial environmental pollution issues [40]. In addition to the natural characteristics of the region, such as aridity, high temperatures, and scarce precipitation, the establishment of manufacturing and power-generating plants have brought consequences in sustainable development [41]. On the one hand, the establishment of the maquiladoras has had a positive impact as a substantial source of manufacturing jobs [42]; however, the weak environmental regulations imposed on these maquiladoras have negatively impacted the quality of water, air, and soil [43]. Mexicali is a remarkable example of this situation. The city is on the northern Mexican border, adjacent to Imperial Valley, California (Figure 1). It has a population of 689,775 inhabitants [44], dry weather and precipitation of around 75 mm/year [45]. One of its leading environmental problems is air pollution [46]. It is ranked as the ninth most polluted place on the American continent due to the high concentration of PM_10_ [4]. Within Mexico, it is the second city with the highest 24-hour average PM_10_ concentration (274 µg/m^3^) just after Torreón city (276 µg/m^3^) [47].

Binational programs have been established through the US–Mexico Border 2020 Program to promote sustainable development in the border regions, including the Mexicali–Imperial Valley region. One of the main objectives is to improve air quality. The programs have advanced in the establishment of guides and strengthening the installed technical capacity. However, agricultural and industrial unsustainable practices, as well as natural sources (mainly erosion caused by wind) have contributed to the deterioration of environmental conditions [48,49]. The study of the PM_10_ chemical composition when the 24-hour average concentration exceeded 120 µg/m^3^ (national index) identified multiple additional sources such as construction activities, burning of wood and household waste, unpaved roads, and dust crushing due to traffic. Those sources share participation in the urban areas at the Mexico–US border [50]. Local strategies to improve air quality in the city are addressed through PROAIRE (“Programa para mejorar la calidad del aire”), which includes scientific and technical information and a set of strategies to reduce the pollution from the identified sources. However, social vulnerability and perception are factors that are not considered in-depth, even though air pollution can disproportionately impact the communities of this area with high migration and poverty rates [51]. Within this context, we explored the social vulnerability and public perception of air quality as relevant factors in local air quality management.

### 2.2. Air Pollution Data and the Exposure Estimation Model

We worked with the concentrations of PM_10_ for 2015, since the data from previous years (2010 to 2015) were statistically insufficient (>25% missing), and in the subsequent years (2016 to 2019), several monitoring stations were out of operation. Thus, the data from 2015 was the most consistent in whole the 2010–2019 range. The data included measurements obtained at the monitoring stations of the National Air Quality Information System (SINAICA) and provided by the Secretaría de Protección al Ambiente, Baja California. We estimated the exposure levels using an interpolation inverse distance weighting (IDW) model. The model used as a base the point data from the monitoring sites. To obtain the possible values of exposure over the space between all the data points, and the visualization of spatial patterns [52], we constructed circular buffers of 5 and 10 km radius. These centered at the monitoring sites and measured the distance to the centroid of each basic geo-statistical area (AGEB by its acronym in Spanish) (Figure S1 in Supplementary Material). AGEBs represent the minimal geographic region where census data is accumulated and reported. The urban area of Mexicali city has 412 AGEBs. Then, we weighted the 24 h average PM_10_ concentrations from monitors that had ≥75% of hourly measurements. In a first interpolation, we assigned the daily AGEB PM_10_ concentration using the IDW raised to the power value of two to those centroids that intersected within 5 km buffers. A second interpolation used the centroids within 10 km buffer intersections for those AGEBs not included in the first, and also raised to the power of two. As a third approach, the centroids outside any 10 km buffer intersection received the PM_10_ concentration of the closest monitoring site. Lastly, for the AGEBs outside the buffers, the IDW raised to the power value of 1 was used, taking as reference the 24 h average concentrations of all the monitoring sites. Téllez-Rojo et al. [53] provided the procedure and validation of this method. We created and used categories based on exceedance days for each AGEB in the subsequent analysis. Exceeded days were those with PM_10_ concentrations above the WHO Air Quality Guideline (AQG) mean value of 50 µg/m^3^ in 24 h. Exposure estimation was obtained for all 412 AGEBs. The analyses were performed using software R, version 3.5.2. (R Foundation for Statistical Computing, Vienna, Austria).

### 2.3. Social Vulnerability Index

We analyzed social vulnerability through socioeconomic status (SES), based on the variables reported in literature linking socioeconomic status and air pollution [15,54,55], as well as data availability. We included variables such as education level, individual economic status (unemployed), health insurance status, women head of household, and access to long lasting consumer goods at the AGEB level from the more recent national population census [44]. The selected variables included the dimension of susceptibility and adaptability, as described by Ge et al. [21]. We used principal components analysis (PCA) to obtain an aggregated value for the range of considered variables. Given that single variables may be autocorrelated, we used the PCA to reduce redundancies and dimensionality. We only included those variables with minimal missing values to minimize the potential for clumping or truncation [56]. We retained the components whose percentage of variance was higher than 10%. Additionally, we used the scree plot based on the eigenvalues proposed by Cattell (1966) [57] (Figure S2 Supplementary Material). The Kaiser–Meyer–Olkin (KMO) index was used as a measure of sampling adequacy (Table S1 Supplementary Material). The index resulting from the PCA analysis was used as a categorical variable based on the standard deviation from the mean, allowing a better visualization of AGEBs with extreme values of social vulnerability. The analysis was performed using Stata version 11.2. (IBM, Armonk, NY, USA).

### 2.4. Spatial Analysis

To analyze the spatial association between PM_10_ exposure and social vulnerability, we used the local bi-variate spatial auto-correlation through Moran’s Index. This indicator quantifies the spatial association between these two variables, the identification of spatial clusters, and their statistical significance [58]. We selected the first order “Queen” spatial weight matrix; this measure of contiguity is based on spatial units that share boundaries and vertices. The variables used to obtain the SES index were not available for the 412 AGEBs; thus, the Queen’s measure was more appropriate. We carried out this spatial analysis using the Geoda 1.12 software (free and open source software) [59].

### 2.5. Data Collection and Statistical Models of Individual Socioeconomic Characteristics and Air Quality Perception

#### 2.5.1. Data Collection from the Survey

We conducted a survey to capture additional individual socioeconomic characteristics and the perception of the air quality among the inhabitants of Mexicali. The survey was part of a contingent valuation study to assess the socioeconomic, perception, and health concerns as potential determinants towards a program of urban afforestation to improve air quality [60]. The questionnaires were applied, face to face, in open spaces (streets, commercial establishments, parks, supermarkets) in January 2019. Pedestrians were randomly selected, and the survey was applied to individuals over 18 years of age and that provided consent. The questions sought to gather quantitative and qualitative information. The items first addressed the demographic and socioeconomic attributes (age, sex, education level, average household income, health insurance, ownership) and the place of residence (street, postal code, and neighborhood) of the respondents. Moreover, the individuals were asked about their concerns for air quality, capturing their perceptions at the local and city level. We used a Likert scale with the categories of very good, good, regular, poor, and very poor air quality. We also explored their perceptions of air quality changes in the last few years (during 2015–2019), presenting them with three scenarios: do you think the air quality in Mexicali has improved; remains unchanged; or worsened? In a third section, we explored another essential aspect, their knowledge about the health effects related to air pollution and pollution sources. We also asked if at least one of the household members presented frequent respiratory symptoms. Two hundred and seventy surveys were applied (sample size has an error type I of 5%). Two inclusion criteria were applied to this sample to select the surveys included in the analysis. First, the respondents must be people with more than four years living in Mexicali, and they must provide some reference for their place of residence. We considered this inclusion criterion essential so that the respondents could provide us with information on the changes observed in recent years. One hundred and ninety-nine surveys were retained. The information about their place of residence allowed us to assign the perception responses to a specific AGEB when the reference was within the polygon. To assess the potential association between air pollution perception and the presence of stationary sources of pollution, we first geocoded the medium-large industries with more than 30 employees and were registered in the National Statistical Directory of Economic Units [61]. We constructed 1.6 km radius buffers centered at the respondent’s place of residence and counted the number of industries within each buffer. The used distance is frequently employed in environmental hazard assessments [62,63]. The assessment of the spatial association between perception and the presence of stationary sources of pollution also used the Queen spatial weight matrix and Moran’s Index. Figure S3 (Supplementary Material) present the buffers used to assess the spatial association between the number of nearby industries and air quality perception.

#### 2.5.2. Statistical Analysis

To identify the association between the estimated variables of exposure and social vulnerability, with the perception of air quality, we used the three categories of the question about the changes perceived in air quality over time (during 2015–2019) as the dependent variable. An ordinal logistic regression model was employed to determine the factors that influenced the perceived changes in the air quality at the individual and neighborhood level. This model is especially relevant when the dependent variable is ordered, in this case:

(1) $$y=\{\begin{array}{c} 1\;improved\\ 2\;unchanged\\ 3\;worsend \end{array}$$

The selection of the included independent variables was based on previous studies [64,65,66,67] and the Kruskal–Wallis H test [68]. The test was used to determine if the dependent variable differs significantly between each group of the independent variables (Table S2, Supplementary Material). The model was as follows:

(2) $$\begin{array}{ll} p_{ij}=\mathrm{Pr}\left(y_{j}=i\right) & =\mathrm{Pr}\left(\mu_{1}<x_{j}\beta +u+\mu_{2}\right)\\ & =\frac{1}{1+\exp\left(-\mu_{2}+x_{j}\beta \right)}-\frac{1}{1+\exp\left(-\mu_{1}+x_{j}\beta \right)} \end{array}$$

where $p_{ij}$ is the change perceived by the individual j, i corresponds to the ordered categories (“improved,” “unchanged,” “worsened”), $x_{j}$ are the independent variables, β are the estimated coefficients, $\mu_{1}$ and $\mu_{2}$ are the thresholds, which divide the ranges of possible values for the dependent variable, for instance, $\mu_{1}$ is the estimated threshold on the ordered variable used to differentiate the “improved” category from the “unchanged” and “worsened” categories when the values of the independent variables were assessed at zero, $\mu_{2}$, is the estimated threshold used to differentiate “improved and “unchanged” categories from “worsened” when the independent variables are evaluated at zero, and u is the error term. The model assumes that the three choices follow the same distribution. Thus, the effects of the independent variables are constant across the choices. This assumption is the well known parallel regression assumption for the proportional odds ratio [69,70] and it was proven in our estimations.

## 3. Results

### 3.1. Estimation and Spatial Distribution of PM_10_ Exposure

**Figure 2:**
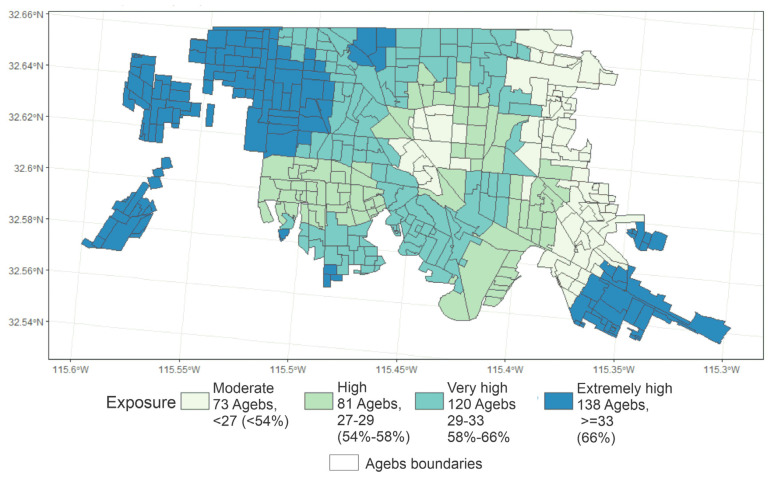
Exposure areas by exceedance days in the urban areas of Mexicali, B.C., at the basic geo-statistical area (AGEB by its acronym in Spanish) level. Parentheses are the percentages of exceedances in days based on 50 days as the maximum measurements available in all the monitoring stations during 2015—categories by quartiles.

The average days with exceedances of the AQG established by the WHO in the AGEBs studied was 60%. Twenty-one days represented the minimum days with exceedances, and 44 days was the maximum. They correspond to 42% and 88% of the days with valid measurements for 2015. The estimated annual median concentration was 77.35 µg/m^3^, the minimum was 59.32 µg/m^3^, and the maximum was 101.93 µg/m^3^. Figure 2 presents the spatial distribution of exposure based on the exceedance days at the AGEBs level. These results show that high concentrations of PM_10_ regularly impacted the general population of Mexicali. AGEBs with higher PM_10_ exposure were located mainly in the West and Southeast regions of the city. These peripheral areas are close to agricultural activities and industrial zones (electric power generation, metallic and nonmetallic mineral products manufacturing, meat processing, and conservation).

### 3.2. Estimation of Social Vulnerability

An initial set of 13 variables from the 2010 Census, including economic, social, and material conditions, were selected, based on the least number of missing values, higher variance, and significance, according to a literature review. The PCA only included six variables (Table 1). This set of variables showed good sampling adequacy (KMO: 0.85).

**Figure 3:**
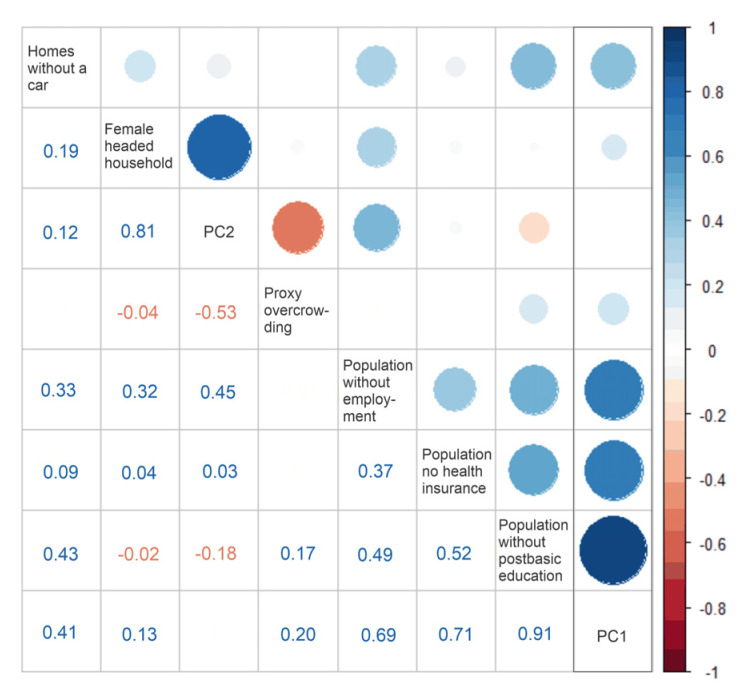
Component loads and correlation among the included variables for principal component 1 (PC1) and principal component 2 (PC2). The blue colors correspond to positive correlations; the red colors correspond to negative correlations. The color intensity and size of the circle are proportional to the component load.

We assigned these variables as proportions to each AGEB before the PCA, due to differences in the population size among AGEBs. Two components with an accumulated variance of 84% were extracted. The first component combined the variables related to social (education, access to health services), and material factors (access to long-lasting consumer goods). Component 2 gave higher weight to financial conditions (unemployment, female-headed households, and overcrowding). Figure 3 shows the correlation of the variables included in the analysis to each one of the selected components.

### 3.3. Spatial Distribution of Social Vulnerability and Autocorrelation with Estimated Exposure to PM_10_

**Figure 4:**
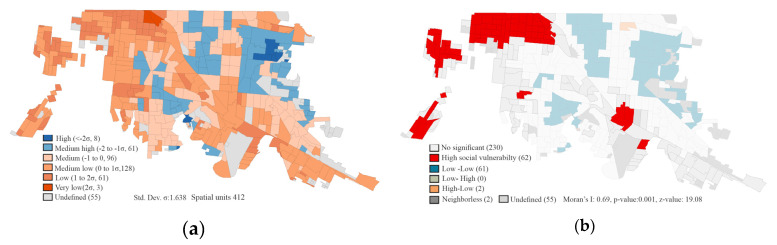

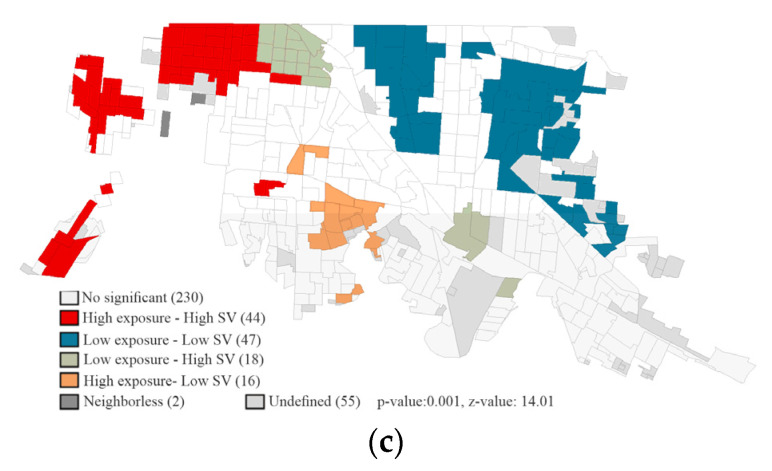
(**a**) Spatial distribution of the socioeconomic status (SES) index. The values in parentheses represent the range by standard deviation and the number of AGEBs for each category. (**b**) Local spatial autocorrelation for the socially vulnerable population. Data from 2010 Census. (**c**) Bivariate local Moran’s Index for spatial autocorrelation between PM_10_ exposure and social vulnerability (SV).

The PCA identified two principal components, Component 1, which includes the variables associated with the susceptibility and adaptive capacity. It also has the statistical power to serve as the SES index used in this study. Figure 4 represents the spatial distribution of the SES index in Mexicali, showing social vulnerability polarization. We identified the hot spots of AGEBs with high social vulnerability and with high levels of PM_10_. The AGEBs with high SES are located in the Northeast region of the city, whereas a higher number of AGEBs with lower SES are located in the Northwest region (Figure 4a). Significant spatial clusters (hotspots) of a socially vulnerable population (Figure 4b) were mainly located in the Northwest and Southeast regions of the city. The distribution from the bivariate local Moran’s Index showed significant spatial clusters of higher vulnerability where higher levels of PM_10_ existed (Figure 4c). Results of the global Moran’s Index were statistically significant (z-score: 18.09 and *p*-value: 0.001). They confirmed that the spatial distribution of high–high follows specific clustering patterns that included 57 AGEBs in the Northwest region of Mexicali. We identified another relevant cluster (five AGEBs) located in the Southeast region with a high level of social vulnerability and an intermediate level of exposure to PM_10_.

### 3.4. Descriptive Statistics and Spatial Distribution of Air Quality Perception

#### 3.4.1. Description of Individual Responses by Air Quality Perceived

**Figure 5:**
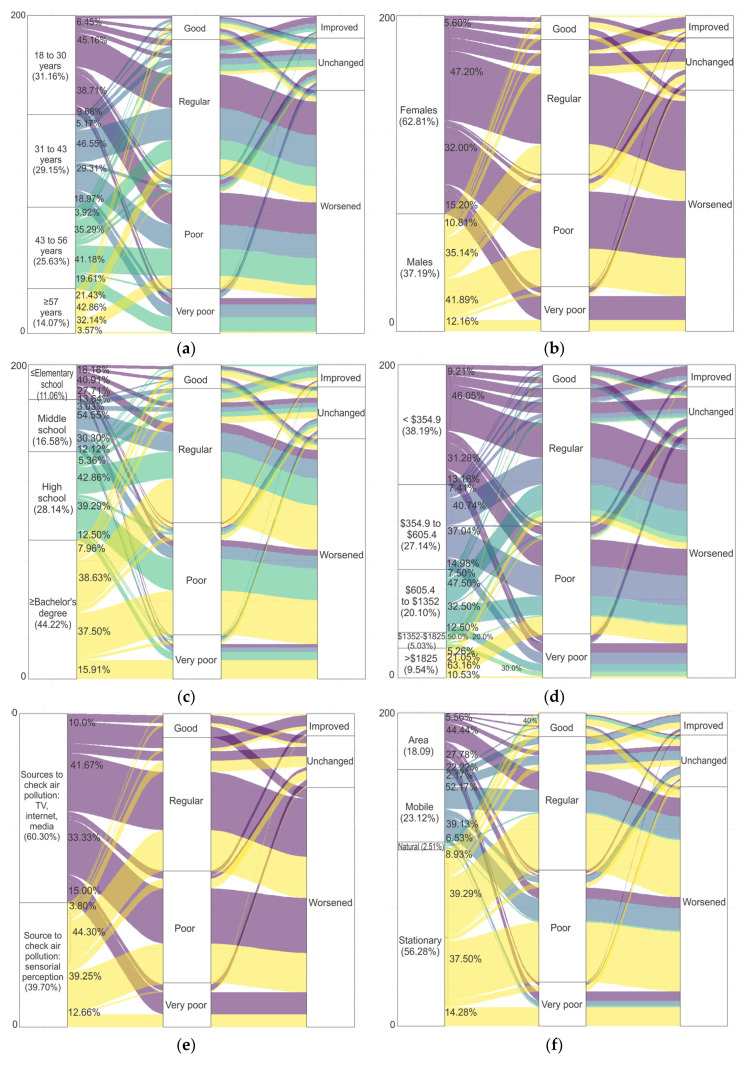

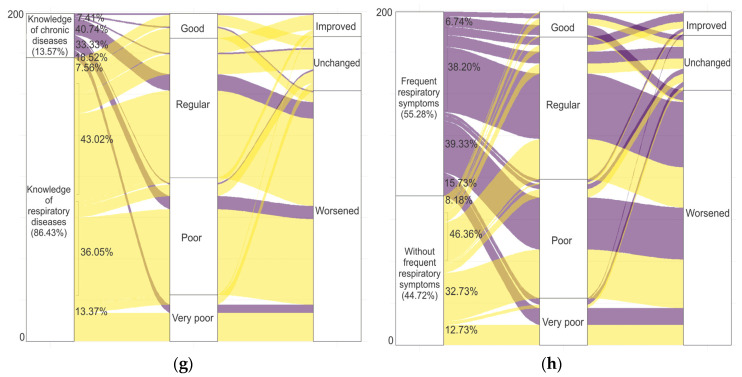
Distribution of perceived air quality at the local level and the perceived changes in the air quality over time by (**a**) age range; (**b**) sex; (**c**) education level; (**d**) average monthly household income (2019 USD); (**e**) sources to check air quality; (**f**) the presence of frequent respiratory symptoms in at least one household member; (**g**) knowledge of the diseases related to air pollution (respiratory or other such as chronic diseases, cancer, neurological and metabolic diseases); and (**h**) the type of pollution sources perceived as more polluting. The colors are related to the number of categories in the first column of each figure. The numbers located next to the first column correspond to the associated percentages of air quality perception at the local level within each category of the first column.

We assessed how the perception of the air quality (city-wide and at the place of residence) diverged by socioeconomic attributes, health status, knowledge of health effects, pollution sources, and the changes perceived in the last four years. Throughout the city, the current air quality was perceived as “regular,” by 32% of the respondents, whereas 46% perceived it as “poor,” and 22% perceived it as “very poor” More than 76% of the respondents perceived that the air quality worsened, whilst 17% considered no change, and 7% perceived that the air quality had improved. We found less consensus at the local level, and some residents even perceived good air quality (around 8%). When the survey was conducted in January 2019, the average concentration of PM_10_ was 87.5 µg/m^3^, and the average annual concentration for that year was 76.75 µg/m^3^, a very close value to the estimated value for 2015. However, we did not use data from 2019, since only one station in the center of the city was monitoring PM_10._ The responses obtained from 199 individuals are in Figure 5. Most respondents were in an age range between 18 and 31 years (31%), and the majority were women (63%) (Figure 5a,b). The highest percentage reported a monthly household income that was less than the II decile of the average nationwide household income [71]. Among the different income levels, the most common perception of air quality was regular or poor, with a perception that it became worse in recent years. The perception of regular or poor air quality had a similar distribution among age groups, while the perception of improved air quality predominated in the young population and women. Similar perceptions occurred at different education levels with a noticeable higher perception of inferior air quality in those individuals with higher education levels (Figure 5c,d). Concerning the way respondents learned about air quality, many of them (60%) reported media such as the Internet, followed by newspapers and TV. A considerable percentage reported only relying on their senses (odors and poor visibility) (40%). This distribution was the same among those who perceived regular or poor air quality. In the case of perceived improvements over time, a higher proportion of respondents learned from the media (Figure 5e). Fifty-six percent of the respondents pointed to stationary sources (manufacturing and generating plants) as the primary sources of air pollution; 23% considered mobile sources; 18% identified area sources (burning of household waste, burning grassland, and unpaved roads); and 3% of respondents considered natural sources such as dust storms (Figure 5f). This distribution did not change in the different air quality categories. However, when exploring the participating sources related to improved air quality over time, a higher proportion of participants identified mobile sources. When inquiring about health concerns and air pollution, 86% mentioned respiratory illness (cough, cold, asthma, allergies), and 14% associated air pollution with chronic diseases, neurological or metabolic conditions. Frequent respiratory symptoms in at least one member in the household was answered by 55% of the respondents (Figure 5g,h). The highest percentage of individuals that perceived air quality worsening over time also reported an association between severe health effects and frequent respiratory symptoms with air pollution. We present the frequencies and percentages associated with each category of perceived air quality at the local level and perceived changes over time in Table S3 of the Supplementary Material.

**Figure 6:**
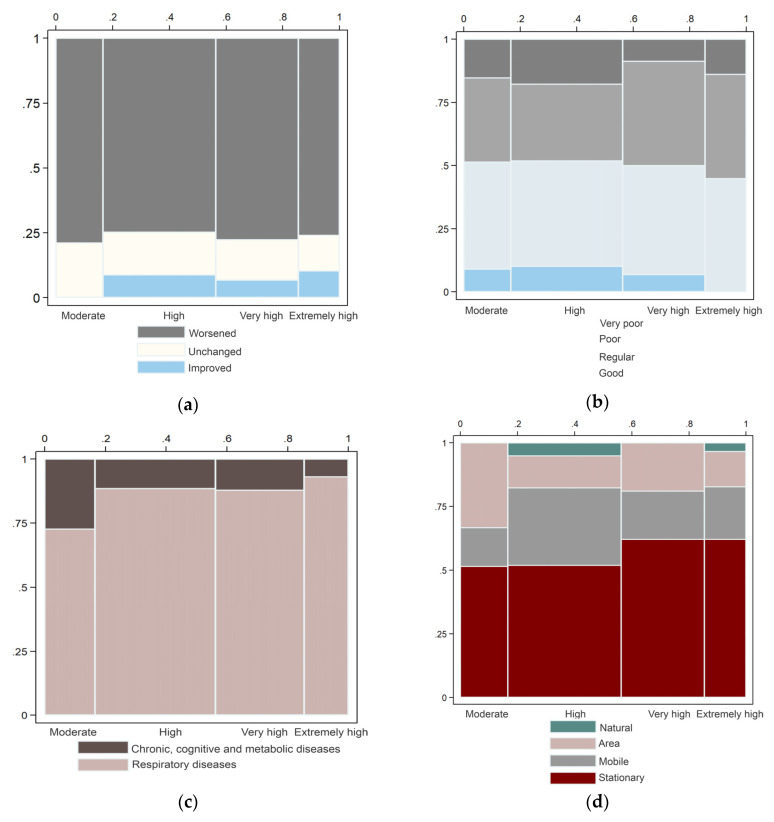
Responses to the perception of air quality by the estimated categories of exposure to PM_10_. (**a**) Fractions of the perceived changes in the air quality on time (last four years); (**b**) fractions of the perceived air quality at a local level; (**c**) fraction of the responses about the knowledge of health effects related to air pollution; and (**d**) fractions of the perceived primary sources of air pollution.

The perceptions of air quality according to the estimated levels of PM_10_ indicated that those respondents in the higher PM_10_ exposure regions perceived, in higher proportions, that the air quality was “poor” or “regular.” In moderately exposed areas, slightly higher proportions perceived the air quality as very poor, and even a small fraction considered it “good.” In general, we identified a limited knowledge of air pollution-related health effects, other than respiratory conditions. A lower proportion of knowledgeable individuals existed in areas of very high exposure. Regarding pollution sources, the stationary sources were the most frequently reported in high exposure areas. Area sources such as agricultural areas, trash burning, and fireworks, particularly in the winter, were frequently identified in those areas with moderate exposure. Figure 6 presents the proportions of responses related to air quality perception, air pollution sources, and the knowledge of air pollution-related health effects in each one of the estimated categories of exposure.

#### 3.4.2. Spatial Distribution of Air Quality Perception, Estimated Exposure to PM_10_ and Stationary Sources of Pollution

**Figure 7:**
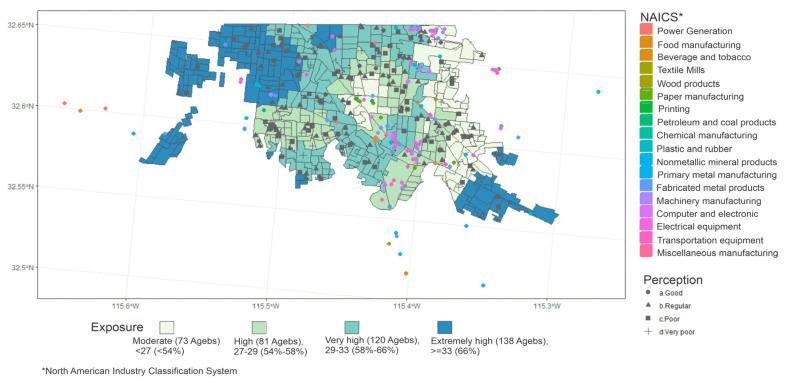
Spatial distribution of the responses about the perception of the current state of air quality, the location of stationary sources of pollution and the estimated categories of exposure to PM_10_ (PM_10_ concentration from the data of monitoring sites from 2015); localized industries correspond to those with more than 30 employees, and that belong to subsectors 221, 311–313, 321–327, 331–336 and 339 according to the North American Industry Classification System.

The geographic distribution of the responses related to perceived air pollution, estimated categories of exposure, and the location of stationary sources of pollution are presented in Figure 7. The perception responses do not show apparent differences among the estimated categories of PM_10_ exposure. A global Moran’s Index for spatial autocorrelation between these two variables does not show a significant association (I: −0.0172, z-score: −0.249, and *p*-value: 0.39). The perception of good air quality was infrequent across the exposure zones. In extremely high exposure zones, we did not find responses of perceived good air quality. However, in the same areas, the perception of very poor air quality was less frequent than the perception categories of “poor” or “regular” air quality. A similar tendency regarding a frequent perception of intermediate categories “regular” and “poor” persists in the remaining exposure level areas. The percentages of responses by the estimated categories of exposure are in Figure S4 (Supplementary Material). Additionally, the geographic distribution of the responses of perceived changes in air quality over time showed a consensus on the perception that the air quality has “worsened” throughout the city (Figure S5, Supplementary Material). Regarding the location of stationary sources of pollution, they are predominantly located at the periphery of the city, mainly in the Southeast region. The western region, which was estimated with high exposure to PM_10_, has electric power generation industries, meat products industries, and metal product manufacturing, which were perceived as very polluting by the residents of these areas. The distribution of responses shows that the proximity to industries was linked to a perception of “very poor” air quality in areas with lower occurrences of PM_10_ exceeding days. The Moran’s Index identified a small cluster where the perception of poor air quality was associated with the high presence of industries near to the respondent’s residence in Southeast Mexicali (I: −0.144, *p*-value: 0.013) (Figure S6, supplementary material).

### 3.5. Statistical Model for the Association between Perceived Air Quality, Socioeconomic Attributes, and Estimated Exposure to PM_10_

Results of the Kruskal–Wallis H test showed significant differences between the categories of average monthly household income and the perceived changes in the air quality of the city. We included the results of the estimated exposure areas at the AGEBs level and the knowledge of health effects related to air pollution in the ordered logistic model as confounding variables based on previous studies [29,65,66]. The estimated coefficients are in Table 2. Results show that income and air quality perception in the place of residence have a significant effect on the perceived air quality changes over time in Mexicali. Respondents who reported low household income had lower odds of perceiving that the air pollution has improved versus the perception of “unchanged” or “worsened” air quality. Respondents who reported that there is “regular poor air quality” in their neighborhood are not likely to report that the air quality has improved in the city. Categories of estimated exposure and social vulnerability included at the AGEBs level did not have a significant effect on the perceived changes. The knowledge on the severity of the health effects related to air pollution does not significantly influence the perception. The small number of respondents who know about the severe health effects may have influenced the coefficients obtained.

Demographic variables such as age, sex, and education level did not have a significant effect on the perceived changes through the ordinal logistic regression model (Table S4, Supplementary Material).

## 4. Discussion

### 4.1. Linking Air Pollution and Social Vulnerability

In this analysis, we assessed social vulnerability, the estimated exposure to PM_10_, measured the public perception of air quality, and evaluated the spatial distribution and the relationships among them. We focused on the importance of the evidence of these links when considering air quality management in the context of sustainable development goals. The estimated PM_10_ levels suggested a high exposure to PM_10_ across the city. We found spatial patterns of higher exposure in AGEBs located in the periphery of the city, specifically in the Northwest and Southeast areas. These areas are near to the sources of PM_10_ emissions and adjacent to agricultural areas, areas with reduced vegetation cover, unpaved roads, and affected by the periodic burning of waste and frequent dust storms [73]. Industry and geothermal plants are contributing to deteriorating the air quality in these areas [74]. When assessing social vulnerability, we found clusters of AGEBs with lower SES located in the same peripheral areas. The SES index indicates that a higher amount of people without post-basic education, with unemployment, and no health insurance reside mainly in the Northwest area, near the Mexico–USA border, and South of Mexicali, where socio-spatial environmental and health inequalities have been reported [36,75,76].

In those peripheral regions, we found clusters (hot spots) of significant spatial autocorrelation between high social vulnerability and high exposure to PM_10_. Therefore, people in these areas have reduced adaptive capacity and have a higher susceptibility to face the risks associated with air pollution [21]; for instance, people with low education levels often access lower-wage jobs. Additionally, in poor working conditions, low-income individuals are often unable to locate homes in places with better environmental conditions or to cover expenses to protect themselves. In Mexico, the “Agenda 2030” for sustainable development [77], particularly Goal 11, proposed that all the public policies require the consideration of the vulnerability to climatic change, especially in disadvantaged populations. However, until now, public policies attending the social vulnerability to the risk associated with air pollution, have not received enough impulse. Evidence of the relationship between socioeconomic drivers and particulate matter pollution comes from other developing countries [78,79]. In those studies, they found relevant links with land use, infrastructure, and energy consumption, that if contemplated, could result in effective policies to reduce socioeconomic disparities [80]. Concerning PM_10_ exposure in Mexicali, we found that the socially deprived population was highly exposed. Urban development programs have been insufficient to reduce potential PM_10_ sources such as unpaved streets or unsustainable agricultural practices. Therefore, the results suggest that it is necessary to align the strategies in the PM_10_ pollution control in Mexicali, as well as urban planning, with social factors in order to promote sustainable development in this region.

### 4.2. The Association of Air Pollution, Socioeconomic Attributes and Air Quality Perception

The spatial distribution of the answers shows that the overall perception is not associated with the modeled exposure to PM_10_. Strong association occurred only in areas with very high exposure. In those areas, air quality perception relies on the individual senses (odors and the visible manifestation of smog and dust). In agreement with previous studies [27,81], we found that perceived changes in air quality occur in areas where people are exposed to the visible manifestation of pollution, such as extremely high concentrations of PM_10_. For instance, a previous analysis of the spatial dimension of environmental and social hazards related to the public perception in Mexicali, Ley-García et al. [82] showed that only 23% of their sample showed congruence between exposure and perception. In those instances, where the perception was higher than the exposure, the proximity to potential sources of pollution played a role. We identified this issue in areas with moderate exposure to PM_10_, where some respondents reported very poor air quality when industries were in the proximity. On the other hand, the sense of attenuation mentioned in [82] is manifested here as well, since only 14% of the respondents living in extremely high exposure areas perceived very poor air quality. This sense of attenuation, in some cases, is related to the order of priorities that people have. Especially in border cities, the violence associated with drugs, immigration, economic and health disparities is part of them. In a similar manner, Mexicali’s industrial growth as a result of a considerable number of manufacturing industries attracted by incentives like cheap labor and the access to a local source of electricity is perceived by the residents as a critical factor of risk. Most of these industries, the settlements where people belonging to lower SES, and air pollutants represent an environmental hazard are concentrated in the peripheral areas of the city. Air quality perception in those areas has an essential role in engaging self-protect actions as well as promoting collective actions to improve environmental conditions.

Our findings from the statistical analysis of low-income level affecting pollution perception (including variables at the individual and community level), agreed with recent studies conducted in the United States, South Korea, and China [29,66]. In our case, when people perceived the surroundings as polluted, they were less likely to consider that the overall air quality in the city improved. The other studies [29,66] reported a similar tendency; lower-income inhabitants had higher odds of considering that the air quality had not improved. These findings also agree with the ones by Schmitz et al. [66], indicating that the concern for pollution, in immediate surroundings, negatively influences the perception of air quality at the city level. Other authors [29,65] postulated that when studying the built environment, individual-level socioeconomic factors are more affordable predictors of air quality perception than objective measurements from monitoring stations. Usually, air quality data is not widely disseminated, and it is not straightforward to interpret [83]. In this regard, the sensitization to engage the population around air pollution problems is necessary to promote behavioral changes towards the air quality control strategies and the self-protection attitudes [34]. Knowledge about the long-term risk of exposure to air pollutants can influence behavioral changes [84]. We found a lack of knowledge about the impact of air pollution on health. Only 7% of the respondents in areas of extremely high exposure were aware of the severity of health effects related to air pollution, particularly in low socioeconomic groups. The knowledge about respiratory diseases, reported by most respondents, is limited to asthma, allergies, cough, and the flu. Other diseases, such as pulmonary cancer and cognitive effects, were not recognized by the respondents. Although existing knowledge of health effects and self-reported diseases related to air pollution have shown a significant association with perceived air quality [63,66], in our study, no statistical significance was obtained. Similar results to ours were obtained in a study conducted in Seoul with a larger sample [29]. A possible reason for this difference is that the population with high social vulnerability and a very high exposure to PM_10_ may be affected by other social problems, limiting their attention to environmental risks such as air pollution [27,80]. Therefore, beyond generating data, governments should provide appropriate communication channels and prioritize actions in the disadvantaged population. Some strategies involve providing understandable information. This goal can be accomplished through media, interventions based on volunteer monitoring and participation with observations, implementing workshops, and using low-cost air quality sensors in low-SES areas. Actions like sustainable agricultural practices, modernizing the supply of conventional energy to clean energy sources, and promoting the planting trees could contribute to increase the wellbeing of this population. Moreover, local governments need to promote source-oriented actions that reduce the emissions of particulate matter from an inclusive perspective. For instance, it is necessary to guarantee the paving of streets in neighborhoods on the periphery where the population is most exposed and lacks the economic resources to do so in a particular way. In Mexico, unpaved streets continue to be a challenge in urban planning; local governments have limited budgets in this area. Hence, it is necessary to show the importance of this issue in cities like Mexicali, which has social, health and mobility implications and the need for prioritized actions.

The objective is to generate an understanding in the population and local officer managers, with a sense of inclusion. 

### 4.3. Limitations

Our study’s limitations are related to the temporary differences among the air quality data obtained for 2015 and the socioeconomic variables from the 2010 Census. These differences may be biased since we used retrospective social vulnerability data; it is not possible to ensure that the individuals remained in the specified AGEBs for the year the exposure was estimated. However, we chose information from 2015 to achieve a higher statistical sufficiency in the air quality records and recent information on socioeconomic characteristics at the AGEB level.

These temporal differences also were also a limitation in the information at the individual level; however, in 2019, only one monitoring site was regularly measuring PM_10_; therefore, it was not possible to estimate exposure at the AGEB level in areas with no functioning monitoring stations. The estimated average annual concentration for 2019 was similar to the one obtained for 2015. In this sense, we considered, as selection criteria, to retain the information, only residents with more than four years living in Mexicali. Although the air quality perception may not adequately reflect the tendencies in air quality if the changes are small, concerning PM_10_, considerable changes may be more noticeable through the senses. Another limitation is related to the small sample size, which may result in un-coverage bias. However, a good spatial coverage was achieved. The sample corresponds to people who visit open spaces with more frequency, and only cover urban areas (see Table S5 in the Supplementary Material to compare population characteristics with the Census data). In this sense, further studies could consider a house-by-house survey.

Despite the technical limitations regarding the availability and coverage of air quality data, our study has an essential contribution in the scarce evidence about the linkages between air pollution, socioeconomic status, and air quality perception in Latin American countries. The findings can serve as a base for further research. Besides, our effort to obtain information in addition to requests from other researchers can put pressure on the consistent generation of data on air quality.

## 5. Conclusions

The results of this study show the existence of spatial patterns of the socially vulnerable population significantly associated with high exposure to PM_10_ in Mexicali. Spatial distribution and statistical analysis indicate that air quality perception at a local level is affecting the lack of perception in air quality changes over time. Considerable agreement between the perception and data from monitoring stations occurred in the areas with a higher exposure to PM_10_; in many cases, the perception of poor or very poor air quality was related to nearby industries. The estimates of PM_10_ exposure and socioeconomic characteristics aggregated at the AGEBs level do not influence the perception in air pollution changes over time. Individual socioeconomic factors such as income have a relevant adverse effect on perceived changes. The knowledge of health effects related to air pollution is scarce in the city, and is not significantly linked to the level of air quality perception. Clusters of the socially vulnerable population living in areas of high exposure to PM_10_ are mostly unaware of the severe health effects associated with air pollution. Therefore, more attention should be directed towards these areas, both in the control of PM_10_-emitting sources and social programs to improve the resilience and adaptive capacity of the residents. Additionally, there is a need for a local risk communication strategy related to the concentrations and the health effects in this part of the city.

The findings of our research contribute to broadening the knowledge about the social factors that affect the exposure and perception of air quality in developing countries. These results may serve as evidence for policymakers to conduct air pollution abatement policies, not only based on technical information but also focused on socioeconomic and cultural factors.

## Figures and Tables

**Table 1:** Description of the socioeconomic variables included in the principal component analysis.

Variables ^1^	*N*	Mean	Std. Dev.	Min	Max
Population without post-basic education ^2^	370	687.43	540.49	1	4290
Population without employment ^3^	357	42.11	30.69	0	168
Population without health insurance (public/private)	370	458.70	301.12	0	1503
Number of households headed by women	370	149.54	100.19	0	470
Number of people per room (overcrowding proxy)	370	3.43	0.51	2	4
Household without a car	370	136.46	115.07	0	750

^1^ The variables are based on metadata defined by National Institute of Statistic, Geography and Informatics (INEGI), 2010 Census [44]. ^2^ People more than 18 years old without post-basic education (bachelor’s degree, master or doctoral studies, complete or incomplete grades). ^3^ People over 12 years old who did not had job, but they looked for a job in the reference week of the census.

**Table 2:** Estimates for the perceived changes in air quality (worsened, unchanged, improved) on time.

Variable	Description	Odds Ratio	95% CI
Average monthly household income (2019 USD) ^1^	<US $354.9 (Ref)		
	From US $354.9 to US $605.4	0.36 **	[0.15–0.90]
	From US $605.4 to US $1352	0.30 **	[0.11–0.86]
	From US $1352 to US $1825	0.42	[0.08–2.20]
	>US $1825	0.48	[0.11–2.02]
Knowledge of health effects related to air pollution ^2^	No (Ref)		
	Yes	0.51	[0.13–1.96]
Perception of air quality in the location of the individual’s residence ^3^	Good (Ref)		
	Regular	0.41	[0.14–1.21]
	Poor	0.21 ***	[0.07–0.69]
	Very poor	0.32 *	[0.08–1.21]
Exposure areas by exceedance days of PM_10_ concentrations ^4^	Moderate (Ref)		
	High	1.22	[0.44–3.39]
	Very high	1.27	[0.43–3.78]
	Extremely high	1.58	[0.42–5.91]
Social vulnerability (categories by SES index) ^5^	Medium-high SES index (Ref)		
	Low SES index	0.58	[0.24–1.38]
*N* = 199 Log-likelihood = −127.54			
Equality coefficient test through the response Chi2(12) = 2.37 Categories ^6^ Pseudo R^2^ = 0.78			

^1^ The exchange rate for when the survey was conducted was 1USD = 19.16 Mexican pesos. World Bank’s collection of development indicators [72]; ^2^ “Yes” if the respondents relate chronic diseases such as cancer, neurological or metabolic diseases with air pollution. ^3^ The place of residence was assigned based on the reference streets or ZIP code. ^4^ The categories of days with exceedances are based on the WHO guideline (50 µg/m^3^). ^5^ The social vulnerability was categorized as very high when the socioeconomic status (SES) index was less than or equal to the 75th centile. ^6^ The result indicates that the proportional odds assumption was not violated. *** for *p* < 0.001, ** *p* < 0.05, * *p* < 0.01.

## Supplementary Materials

The following are available online at https://www.mdpi.com/1660-4601/17/13/4616/s1, Figure S1: Geographic location of monitoring stations, centroids of each AGEB and buffers used for the IDW method, Figure S2: Principal components analysis scree plot as selection criteria, Figure S3: Circular buffers to assess proximity to industries and perception of air quality. 1-mile radius buffers were centered at the respondent’s place of residence. The number of industries within each buffer was calculated. We use the “queen” proximity matrix, Figure S4: Responses of perceived air quality by exposure areas, Figure S5: Perceived changes on air quality in the last four years in Mexicali. Survey 2019, Figure S6: Bivariate local Moran’s Index for spatial autocorrelation between the presence of industries nearby the residence’s place and perception of air quality. Localized industries correspond to those with more than 30 employees, and that belong to subsectors 221, 311–313, 321–327, 331–336 and 339 according to the North American Industry Classification System, Table S1: Results of the Kaiser-Meyer-Olkin (KMO) for measuring adequacy sampling, Table S2: Results of the Kruskal-Wallis H test for individual and AGEB level variables with responses of air quality perception. Consist of the chi-square test statistic with ties (Χ2), the degrees of freedom (df) and the significance level (p-value), Table S3: Socioeconomic characteristics, health concerns and knowledge of health effects by the perceived air quality, Mexicali, 2019. Percentages of responses in parenthesis, Table S4: Estimates for perceived changes in air quality (worsened, unchanged, improved), Table S5: Demographic characteristics of the sample and information from the 2015 Census for the municipality of Mexicali.
