# Supplementary material for: Assessing the Influence of Socioeconomic Status and Air Pollution Levels on the Public Perception of Local Air Quality in a Mexico-US Border City
Source: Int J Environ Res Public Health. 2020 Jun 27;17(13):4616. doi: 10.3390/ijerph17134616 (PMC7369924; doi:10.3390/ijerph17134616)
Supplement: Supplementary file 1 [file ijerph-17-04616-s001.pdf]

International Journal of
Environmental Research and Public Health

MDPI

Supplementary Material

Assessing the influence of socioeconomic status and air pollution levels on the public perception of local air quality in a Mexico-US border city.

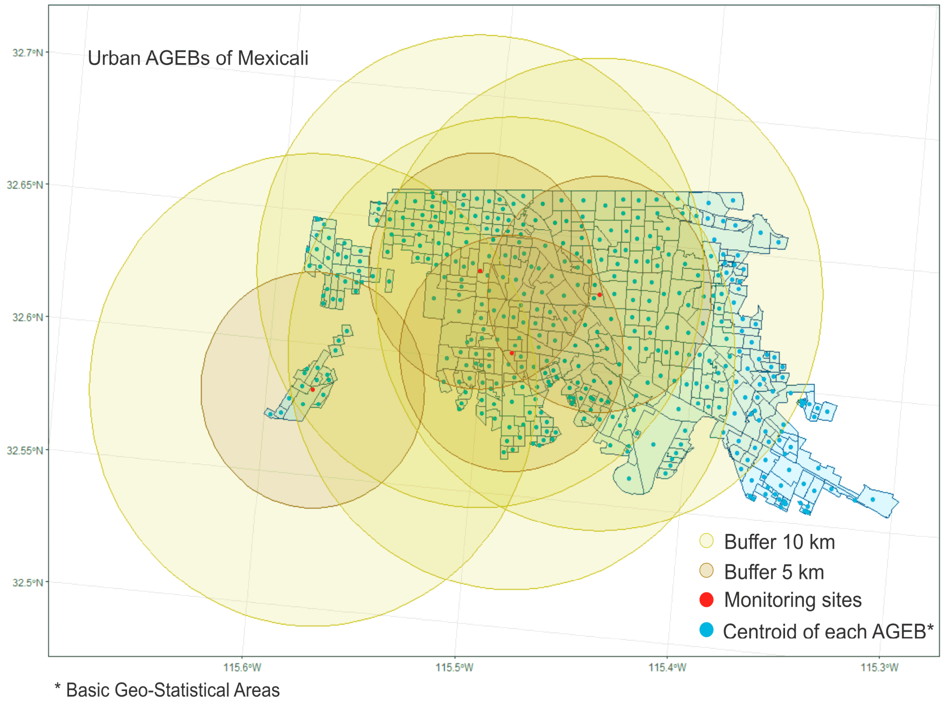

Figure S1. Geographic location of monitoring stations, centroids of each AGEB and buffers used for the IDW method.

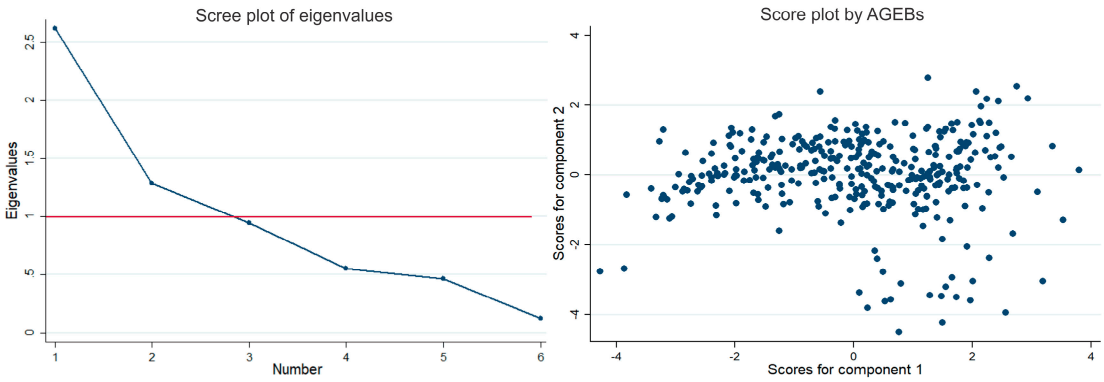

Figure S2. Principal components analysis scree plot as selection criteria.

Table S1. Results of the Kaiser-Meyer-Olkin (KMO) for measuring adequacy sampling.

**Variables**	**KMO**
Pop. >18 years of age without post-basic education	0.87
Pop. >18 years of age without employment	0.94
Pop. Without health insurance (public/private)	0.83
Number of households headed by women	0.82
Number of people per room (overcrowding proxy)	0.42
Households without a car	0.83
Overall	0.85

Table S2. Results of the Kruskal-Wallis H test for individual and AGEB level variables with responses of air quality perception. Consist of the chi-square test statistic with ties (X^2), the degrees of freedom (df) and the significance level (p-value).

Variable	X^2 with ties[a]	df	p-value
Age (Four categories: 18 to 30 years old, 31 to 43 years old, 44 to 56 years old, more than 57 years old)	0.262	3	0.967
Sex (Females and males)	1.216	1	0.270
Education level (Lower or equal to elementary school, middle school, high school and higher or equal to bachelor's degree)	2.904	4	0.574
Average household monthly income (Five categories)	9.575	4	0.048*
Health insurance status (Two categories: public/private and without health insurance)	2.598	1	0.107
Sources to check air quality (Media and visible signs of pollution)	0.088	1	0.766
Frequent respiratory symptoms in at least one household member (Affected and non-affected household members)	0.349	1	0.554
Knowledge of health effects related to air pollution (only respiratory diseases and report of respiratory diseases, cancer, neurological and metabolic diseases)	2.615	1	0.105
Sources of pollution (Four categories: fixed, mobile, area sources and natural sources)	0.312	3	0.701
Perception at the neighbourhood level	9.308	3	0.025*
Estimated exposure areas by exceedance in days (Four categories: moderate, high, very high, extremely high)	0.513	3	0.916

[a] Kruskal-Wallis H test is a non-parametric statistical approach used to compare differences between two or more groups of an independent variables on an ordered dependent variable. The test does not assume population normality, nor homogeneity of variance.

*A statistically significant difference in perception between the categories of the independent variable at 0.05

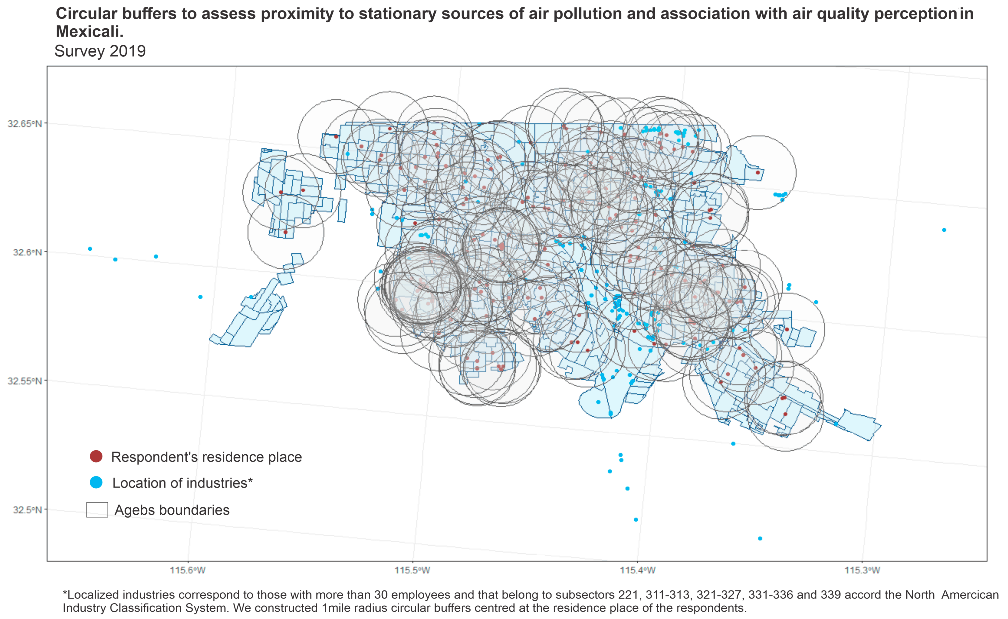

Figure S3. Circular buffers to assess proximity to industries and perception of air quality. 1-mile radius buffers were centered at the respondent`s place of residence. The number of industries within each buffer was calculated. We use the "queen" proximity matrix.

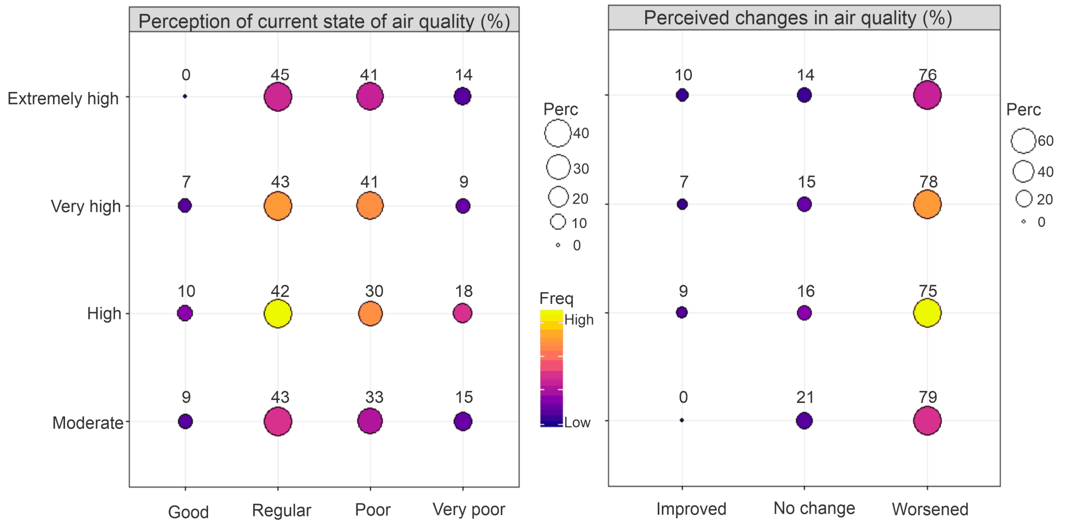

Figure S4. Responses of perceived air quality by exposure areas.

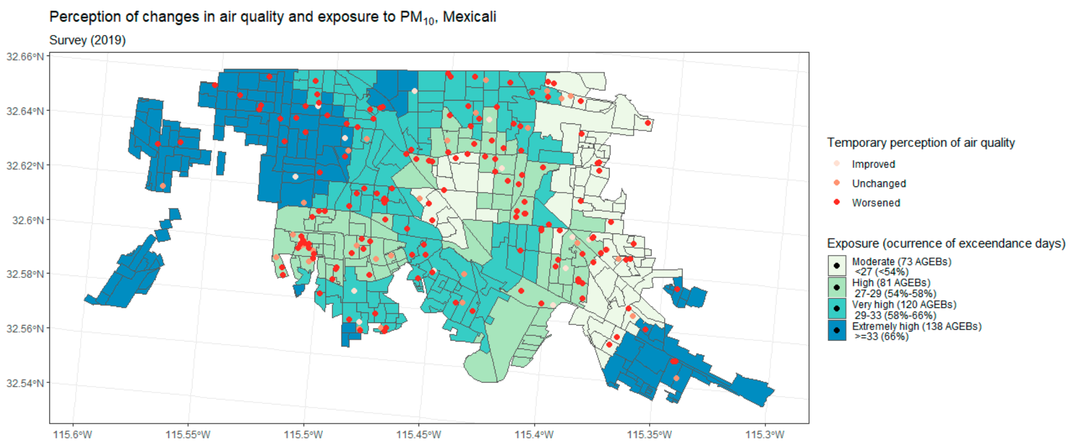

Figure S5. Perceived changes on air quality in the last four years in Mexicali. Survey 2019.

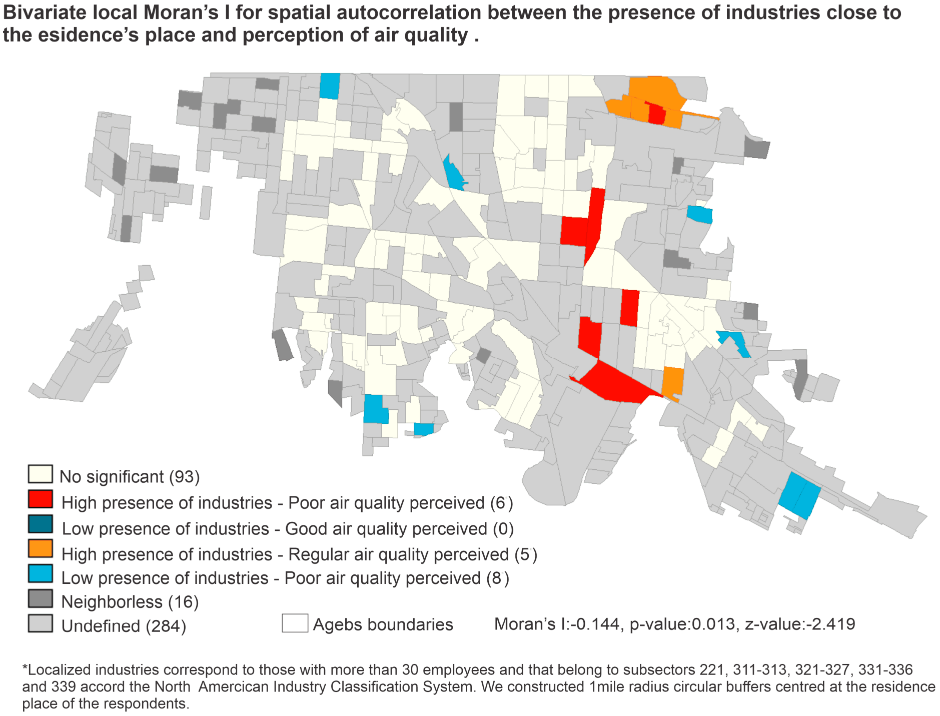

Figure S6. Bivariate local Moran's Index for spatial autocorrelation between the presence of industries nearby the residence's place and perception of air quality. Localized industries correspond to those with more than 30 employees, and that belong to subsectors 221, 311-313, 321-327, 331-336 and 339 according to the North American Industry Classification System.

Table S3. Socioeconomic characteristics, health concerns and knowledge of health effects by the perceived air quality, Mexicali, 2019. Percentages of responses in parenthesis.

Perception of air quality N=199								
	Improved (I), Unchanged (U), Worsened (W)			Good (1), Regular (2), Poor (3), Very poor (4)				
**Variables**	**I**	**U**	**W**	**1**	**2**	**3**	**4**	**Total**
**Age**								
18 to 30 years old	6 (9.68)	9 (14.52)	47 (75.81)	4 (6.45)	28 (45.16)	24 (38.71)	6 (9.68)	62 (31.16)
31 to 43 years old	3 (5.17)	11 (18.97)	44 (75.86)	3 (5.17)	27 (46.55)	17 (29.31)	11 (18.97)	58 (29.15)
44 to 56 years old	5 (9.80)	7 (13.73)	39 (76.47)	2 (3.92)	18 (35.29)	21 (41.18)	10 (19.61)	51 (25.63)
≥57 years old	0 (0.00)	6 (21.43)	22 (78.57)	6 (21.43)	12 (42.86)	9 (32.14)	1 (3.57)	28 (14.07)
**Sex**								
Males	5 (6.76)	16 (21.62)	53 (72.62)	8 (10.81)	26 (35.14)	31 (41.89)	9 (12.16)	74 (37.19)
Females	9 (7.90)	17 (13.60)	99 (79.20)	7 (5.60)	59 (47.2)	40 (32.00)	19 (15.20)	125 (62.81)
**Education level**								
≤Elementary school	1 (4.55)	7 (31.81)	14 (63.64)	4 (18.18)	9 (40.91)	6 (27.71)	3 (13.64)	22 (11.06)
Middle school	3 (9.09)	6 (18.18)	24 (72.73)	1 (3.03)	18 (54.55)	10 (30.30)	4 (12.12)	33 (16.58)
High school	6 (10.71)	7 (12.50)	43 (76.79)	3 (5.36)	24 (42.86)	22 (39.29)	7 (12.50)	56 (28.14)
≥Bachelor's degree	4 (4.55)	13 (14.77)	71 (80.68)	7 (7.96)	34 (38.63)	33 (37.50)	14 (15.91)	88 (44.22)
**Average monthly household income (2019 USD)**								
< $354.9	8 (10.53)	19 (25.00)	49 (64.47)	7 (9.21)	35 (46.05)	24 (31.28)	10 (13.16)	76 (38.19)
From $354.9 to $605.4	4 (7.41)	5 (9.26)	45 (83.33)	4 (7.41)	22 (40.74)	20 (37.04)	8 (14.81)	54 (27.14)
From $605.4 to $1352	1 (2.50)	5 (12.50)	34 (85.00)	3 (7.50)	19 (47.50)	13 (32.50)	5 (12.50)	40 (20.10)
From $1352 to $1825	0 (0.00)	2 (20.00)	8 (80.00)	0 (0.00)	5 (50.00)	2 (20.00)	3 (30.00)	10 (5.03)
>$1825	1 (5.26)	2 (10.53)	16 (84.21)	1 (5.26)	4 (21.05)	12 (63.16)	2 (10.53)	19 (9.55)
**Health insurance (public or private)**								
With health insurance	11 (6.43)	26 (15.20)	134 (78.36)	11 (6.43)	73 (42.69)	64 (37.43)	23 (13.45)	171 (85.93)
Without health ins.	3 (10.71)	7 (25.00)	18 (64.29)	4 (14.29)	12 (42.89)	7 (25.00)	5 (17.86)	28 (14.07)
**Sources to check air quality**								

TV, internet, newspaper	10 (8.33)	17 (14.17)	93 (77.50)	12 (10.00)	50 (41.67)	40 (33.33)	18 (15.00)	120 (60.30)
Sensorial perception	4 (5.06)	16 (20.25)	59 (74.68)	3 (3.80)	35 (44.30)	31 (39.24)	10 (12.66)	79 (39.70)
**Frequent respiratory symptoms in at least one household member**								
Yes	6 (6.74)	17 (19.10)	66 (74.16)	6 (6.74)	34 (38.20)	35 (39.33)	14 (15.73)	110 (55.28)
No	8 (7.27)	16 (14.55)	86 (78.18)	9 (8.18)	51 (46.36)	36 (32.73)	14 (12.73)	89 (44.72)
**Knowledge of health diseases related to air pollution** (report respiratory diseases or other such as chronic diseases, cancer, neurological and metabolic diseases)								
Respiratory diseases	13 (7.56)	31 (18.02)	128 (74.42)	13 (7.56)	74 (43.02)	62 (36.05)	23 (13.37)	172 (86.43)
Chronic diseases	1 (3.70)	2 (7.41)	24 (88.89)	2 (7.41)	11 (40.74)	9 (33.33)	5 (18.52)	27 (13.57)
**Pollution sources**								
[1]Stationary	6 (5.36)	15 (13.39)	91 (81.25)	10 (8.93)	44 (39.29)	42 (37.50)	16 (14.29)	112 (56.28)
[2]Mobile	32 (69.57)	10 (21.74)	4 (8.70)	1 (2.17)	24 (52.17)	18 (39.13)	3 (6.53)	46 (23.12)
[3]Area	26 (72.22)	6 (16.67)	4 (28.57)	2 (5.56)	16 (44.44)	10 (27.78)	8 (22.22)	36 (18.09)
[4]Natural	3 (60.00)	2 (40.00)	0 (0.00)	2 (40.00)	1 (20.00)	1 (20.00)	1 (20.00)	5 (2.51)
**Total**	14 (7.04)	33 (16.58)	152 (76.38)	15 (7.54)	85 (42.71)	71 (35.68)	28 (14.07)	199

[1]Stationary source: electric power generation, food processing plants and heavy industrial sources. [2]Mobile source: automobiles, trucks, buses. [3]Area source: commercial and services establishments, dry cleaners, gas stations, agricultural areas, unpaved roads, burning of household waste, fireworks. [4]Natural: dust storms, wind erosion, wildfires.

Table S4. Estimates for perceived changes in air quality (worsened, unchanged, improved).

		Odds-ratio and 95% Confidence interval			
**Variable**	**Description**	**Socio-economic attributes**	**Knowledge of health effects**	**Local perception**	**Var. at the AGEB level**
Average monthly household income (2019 USD)[a]	< US $354.9 (Ref)				
	From US $354.9 to US $605.4	0.39** [0.16-0.97]	0.39** [0.17-0.93]	0.40** [0.17-0.97]	0.36** [0.15-0.90]
	From US $605.4 to US $1352	0.28** [0.09-0.89]	0.36** [0.13-0.97]	0.35** [0.13-0.95]	0.30** [0.11-0.85]
	From US $1352 to US $1825	0.32 [0.05-1.92]	0.44 [0.09-2.20]	0.47 [0.09-2.41]	0.42 [0.08-2.20]
	> US $1825	0.28 [0.06-1.30]	0.43 [0.11-1.67]	0.53 [0.13-2.18]	0.47 [0.11-2.02]
Education level	≤ Elementary school (Ref)				
	Middle school	0.83 [0.25-2.75]			
	High school	0.67 [0.20-2.22]			
	Bachelor's degree	0.82 [0.24-2.85]			
	Graduate school	1.41 [0.26-7.73]			
Sex	Male (Ref)				
	Female	0.69 [0.34-1.39]			
Age	18 to 30 years old (Ref)				
	31 to 43 years old	0.76 [0.31-1.86]			
	44 to 56 years old	0.87 [0.32-2.35]			
	≥57 years old	0.51 [0.15-1.73]			
[b] Knowledge of health effects related to air pollution	No (Ref)				
	Yes		0.50 [0.13-1.85]	0.48 [0.13-1.84]	0.51 [0.13-1.95]
Perception of air quality in the location of the individual's residence	Good (Ref)				
	Regular			0.42 [0.14-1.23]	0.41 [0.14-1.21]
	Poor			0.21*** [0.07-0.67]	0.21*** [0.07-0.68]
	Very poor			0.30* [0.08-1.14]	0.32* [0.08-1.21]
	Moderate (Ref)				
	High				1.22 [0.44-3.39]

[c] Exposure areas by exceedance days of PM_{10} concentrations	Very high				1.27 [0.43-3.78]
	Extremely high				1.58 [0.42-5.91]
[d] Social vulnerability (through SES index)	Medium-High SES index (Ref)				
	Low SES index				0.58 [0.24-1.37]
N=199		LLV= -131.29	LLV=-132.09	LLV=-128.44	LLV=-127.54
		LrChi2=0.43	LrChi2=0.05	LrChi2=0.03	LrChi2=0.07
[e] Equality coefficient test through the response categories		Chi2(12)=2.08 Prob>chi2=0.72	Chi2(5)=0.17 Prob>chi2=0.92	Chi2(8)=0.14 Prob>chi2=0.98	Chi2(12)=2.37 Prob>chi2=0.79

[a] The exchange rate for when the survey was conducted was 1 USD = 19.16 Mexican pesos. World Bank's collection of development indicators (WDI, 2019).

[b] "Yes" if respondents relate chronic diseases such as cancer, neurological or metabolic diseases with air pollution

[c] The categories of days with exceedances are based on the WHO guideline (50 µg m^{-3})

[d] Social vulnerability was categorized as very high when the SES index was less than or equal to 75th centile.

[e] This test is a likelihood ratio test for ordinal response models proposed by Wolfe and Gould (1998), the result indicates that the proportional odds assumption was no violated.

*** for p<0.001, **p<0.05, *p<0.01

Table S5. Demographic characteristics of the sample and information from the 2015 Census for the municipality of Mexicali.

**Variables**	**Sample N (%)**	**Total Mexicali, 2015 Census (%)**
**Age***		
19 to 29 years old	56 (28.14)	170,041 (26.43)
30 to 44 years old	71 (35.68)	222,159 (34.54)
45 to 59 years old	53 (26.63)	94,665 (14.71)
≥60 years old	19 (9.55)	95,121 (14.78)

**Sex**		
Males	74 (37.19)	317,310 (49.35)
Females	125 (62.81)	325,623 (50.65)
**Education level***		
≤Elementary school	22 (11.06)	155,822 (21.36)
Middle school	33 (16.58)	231,325 (31.71)
High school	56 (28.14)	181,281 (24.85)
≥Bachelor's degree	88 (44.22)	159,688 (21.89)

* The categories definitions are different from the ones in the 2015 Census. We considered the population with a technical degree in the higher category. The remaining 0.19 in the percentages of the census corresponds to a population that does not specified their education level.
